# Health in Prisons

**Published:** 1871-07

**Authors:** 


### HEALTH IN PRISONS.

"His health has improved remarkably since his imprisonment," is the statement made of a man under sentence of death in New Jersey for setting a ship on fire at sea. He was respectably connected, a man of intelligence, and in the prime of life. Here is a man sent to jail sick; accustomed to an out-door life; and yet with all the anxieties about his trial for months, and then the daily, hourly contemplation of a horrible and disgraceful death on the gallows, his health steadily improves. It is an undisputed fact that "prison life" "agrees" with multitudes in spite of all the mental and moral disturbances in association; it is because prison fare is plain, regular, and moderate; prisoners go to bed early, have abundant time for rest, and for the most part no opportunities are afforded for indulgence in liquors, tobacco, and degrading vices.

An observant army officer said, during the war, that the wounded more generally got well if they remained in camp; while the majority of those sent home to be nursed by friends and kindred were pretty sure to die. He attributed this unexpected result to the fact that those who were sent home were so pampered in their eating, by the sympathy and mistaken judgment of their friends, that the stomach was overpowered, and could not get the necessary strength out of their food to throw off disease. These facts clearly and strongly prove that plain, nourishing food, with regular habits of eating and sleep, are of greater value in the restoration of health from ordinary diseases than unprofessional persons are aware of; and if plain, regular living, under depressing circumstances, has such a powerful instrumentality in recovering from disease, we may reasonably expect much greater and better things in the direction of the maintenance of health to a good old age in the practice of a temperate and regular life.

